# Supplementary material for: Evidence for the Presence of Borrelia burgdorferi Biofilm in Infected Mouse Heart Tissues
Source: Microorganisms. 2024 Aug 26;12(9):1766. doi: 10.3390/microorganisms12091766 (PMC11434270; doi:10.3390/microorganisms12091766)
Supplement: Supplementary file 1 [file microorganisms-12-01766-s001.zip › microorganisms-3152371-supplementary.pdf]

Supplementary Figures

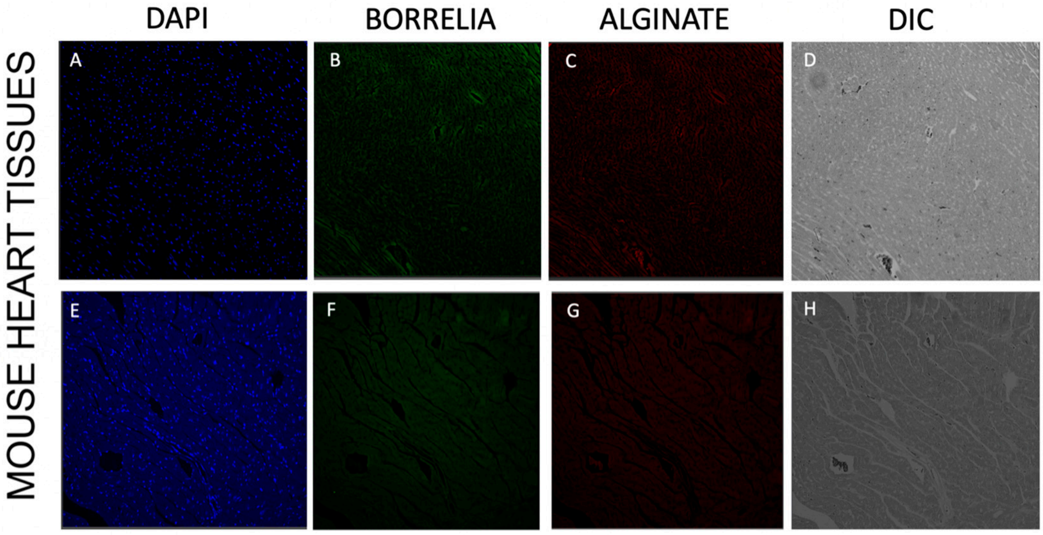

Figure S1. Representative images of IHC staining of normal mouse heart tissues. Panels (**A**,**E**) show the DAPI staining for nuclear DNA. Panels (**B**,**F**) show the absence of *B. burgdorferi*-specific staining while Panels (**C**,**G**) show the absence of alginate-specific staining on those normal mouse heart sections. Panels (**D**,**H**) show the structure of the tissue by differential interference contrast microscopy (DIC). Images were taken at 400×.

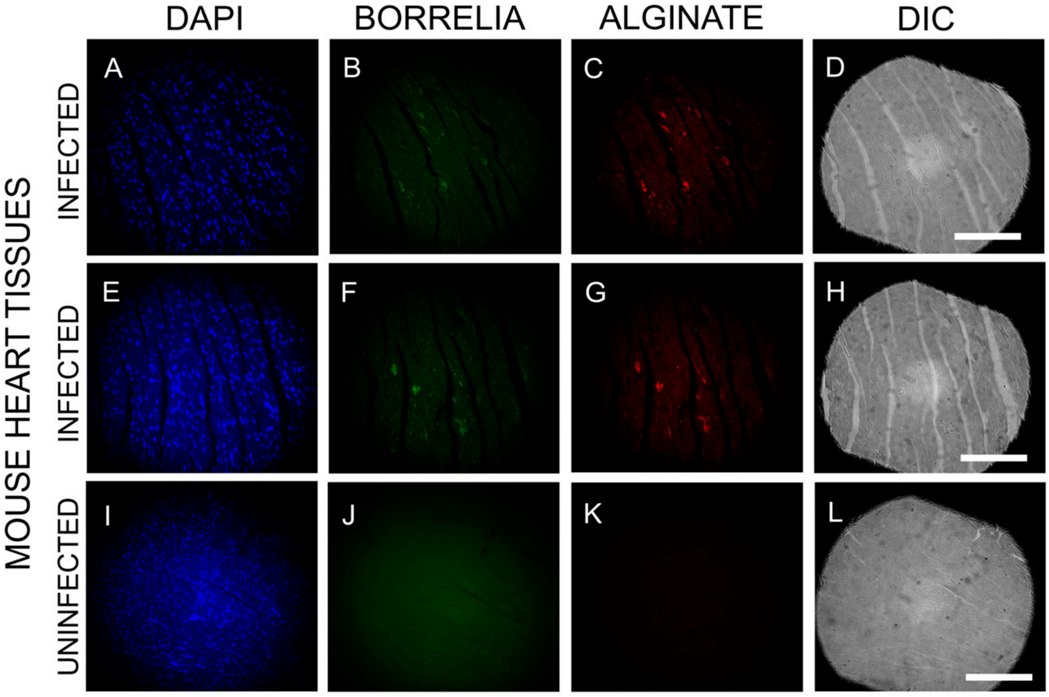

Figure S2. Detection of *B. burgdorferi* biofilm in heart tissue sections of C3H/HeN infected and uninfected mice. Panels (**B**,**F**) show results using an anti-*B. burgdorferi* antibody (green). Panels (**C**,**G**) show results using an anti-alginate antibody (red). Panels (**A**,**E**,**I**) show DAPI staining for nuclear DNA. Panels (**J**,**K**) show negative control, uninfected mouse heart sections that were stained with the same *Borrelia* and alginate antibodies. Panels (**D**,**H**,**L**) show the structure of the tissues by DIC. Images were taken at 200×. Bar: 200 μm.

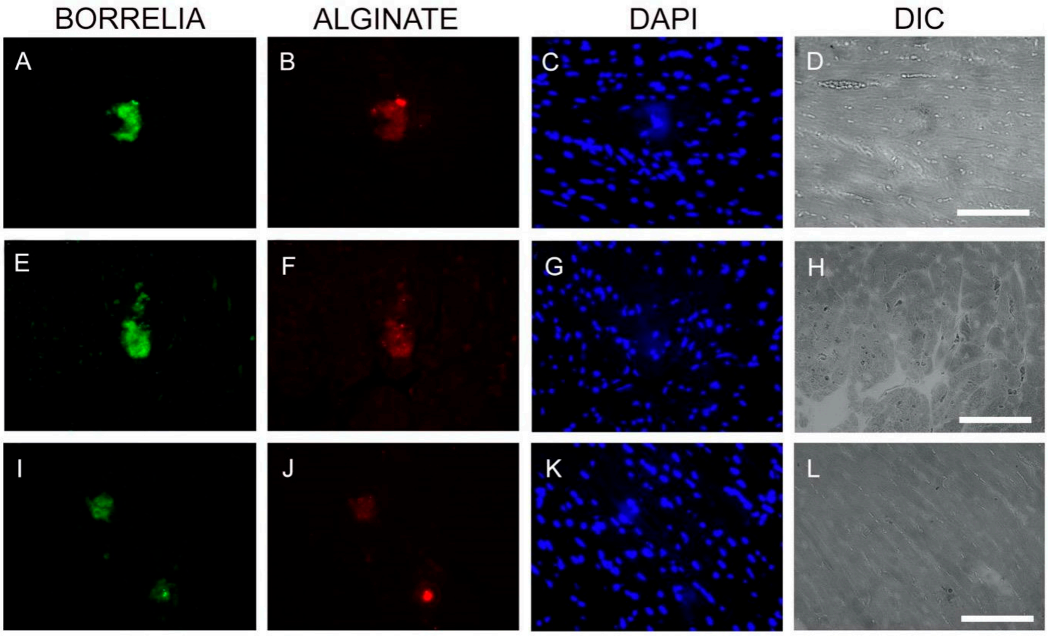

Figure S3. Detection of *B. burgdorferi* biofilm in heart tissue sections of C3H/HeN infected mice. Panels (**A**,**E**,**I**) show results using an anti-*B. burgdorferi* antibody (green). Panels (**B**,**F**,**J**) show results using an anti-alginate antibody (red). Panels (**C**,**G**,**K**) show DAPI staining for nuclear DNA. Panels (**D**,**H**,**L**) show the structure of the tissues by DIC. Images were taken at 400×. Bar: 100 μm.
